# Supplementary material for: An open-source framework for large-scale, flexible evaluation of biomedical text mining systems
Source: J Biomed Discov Collab. 2008 Jan 29;3:1. doi: 10.1186/1747-5333-3-1 (PMC2276192; doi:10.1186/1747-5333-3-1)
Supplement: Additional file 1 — Evaluation platform source code. The additional file contains the software evaluation framework discussed in this paper. The most current release can be downloaded from SourceForge.net [1]. See the accompanying README file for instructions on installation and use. Also included in the distribution is a collection of UIMA wrappers for some commonly used BioNLP tools and annotated corpora. [file 1747-5333-3-1-S1.zip › bionlp_uima_repository-1.0.1/docs/api/src-html/edu/uchsc/ccp/util/nlp/annotation/printer/DefaultPrintOutput.html]

```
001    package edu.uchsc.ccp.util.nlp.annotation.printer;
002    
003    import java.io.PrintStream;
004    import java.util.HashMap;
005    import java.util.List;
006    
007    import edu.uchsc.ccp.util.nlp.annotation.TextAnnotation;
008    import edu.uchsc.ccp.util.nlp.document.DocumentSection;
009    import edu.uchsc.ccp.util.nlp.document.DocumentUtil;
010    import edu.uchsc.ccp.util.nlp.document.GenericDocument;
011    
012    public class DefaultPrintOutput implements AnnotationPrinterUtil {
013        private boolean useOneLineFormat = false;
014        
015        public void printDocumentCollection(List<GenericDocument> documents) {
016            printDocumentCollection(documents, System.out);
017        }
018        
019        public void printDocumentCollection(List<GenericDocument> documents, PrintStream ps) {
020            for (GenericDocument gd : documents) {
021                printDocument(gd, ps);
022            }
023        }
024        
025        public void printDocument(GenericDocument gd) {
026            printDocument(gd, System.out);
027        }
028        
029        public void setUseOneLineFormatStatus(boolean useOneLineFormat) {
030            this.useOneLineFormat = useOneLineFormat;
031        }
032        
033        public void printDocument(GenericDocument gd, PrintStream ps) {
034            List<TextAnnotation> annotations = gd.getAnnotations();
035            
036            ps.println("==============================================================================");
037            // print document information
038            String docID = gd.getDocumentID();
039            int docSize = gd.getDocumentText().length();
040            ps.println("DOCUMENT ID: " + docID);
041    
042            // print document section information
043            HashMap<Integer, String> docSectionIDNameHash = DocumentUtil.getSectionIDNameHash();
044            List<DocumentSection> documentSections = gd.getDocumentSections();
045            String sections = "";
046            if (documentSections != null) {
047            for (DocumentSection ds : documentSections) {
048                sections += (docSectionIDNameHash.get(ds.getDocumentSectionID()) + "(" + (ds.getSectionEndIndex()-ds.getSectionStartIndex()) + ")  ");
049            }
050            }
051            
052            ps.println("DOCUMENT LENGTH: " + docSize);
053            ps.println("DOCUMENT SECTIONS: " + sections);
054            ps.println("DOCUMENT: " + gd.getDocumentText());
055    
056            // iterate and print annotations
057            ps.println("------------------------------------------------------------------------------------");
058            ps.println("------------------------------      ANNOTATIONS      -------------------------------");
059            ps.println("------------------------------------------------------------------------------------");
060    
061            for (TextAnnotation ta : annotations) {
062                if (useOneLineFormat) {
063                        ta.printAnnotationOnOneLine(ps);
064                } else {
065                    ta.printAnnotation(ps);
066                }
067            }
068            ps.println("==============================================================================");
069            
070        }
071        
072        
073    }
```
